# Nutritional Status and Malnutrition-Associated Factors Among Patients Undergoing Hemodialysis and Peritoneal Dialysis: A Cross-Sectional Single-Center Study in Jeddah, Saudi Arabia

**DOI:** 10.7759/cureus.98304

**Published:** 2025-12-02

**Authors:** Arwa M Alsubaie, Mehad M Alrewaithi, Nouf Bin Jahlan, Bedoor K Alghamdi, Yara S Alamro, Salem H Al-Qurashi

**Affiliations:** 1 Nutrition, King Fahad Armed Forces Hospital, Jeddah, SAU; 2 Nutrition, Faculty of Applied Medical Science, King Saud University, Riyadh, SAU; 3 Renal Diseases and Transplantation Centre, King Fahad Armed Forces Hospital, Jeddah, SAU

**Keywords:** biochemical data, end-stage renal disease, hand grip strength, hemodialysis, malnutrition, modified subjective global assessment, peritoneal dialysis

## Abstract

Introduction: Malnutrition and protein-energy wasting (PEW) are significant challenges for patients undergoing dialysis, whether they receive hemodialysis (HD) or peritoneal dialysis (PD). These conditions are strongly linked to a decreased quality of life and an increased risk of illness and death.

Methods: This cross-sectional single-center observational study aimed to comprehensively evaluate the nutritional status of HD and PD patients and investigate contributing factors at the dialysis center of the King Fahad Armed Forces Hospital in Jeddah, Saudi Arabia. Malnutrition was assessed using the Dialysis Malnutrition Score (DMS), which is the modified subjective global assessment (M-SGA). The DMS includes detailed medical history and physical examination. Other patient characteristics, such as sociodemographic features, health status, hand grip strength, 24-hour dietary recall, and biochemical measurements, were also determined. Two hundred (81.6%) patients were undergoing HD, and 45 (18.4%) were undergoing PD.

Result: Of the 245 patients, 233 (95.10%) had mild malnutrition and five (2.04%) had moderate malnutrition. None of the patients had severe malnutrition. PD (P = 0.023, 95% CI 0.23-0.307), female gender (P < 0.001, 95% CI -2.95 to -0.93), number of dialysis years (P = 0.001, 95% CI 0.07-0.24), average hand grip strength (P < 0.001, 95% CI -0.10 to -0.03), and average skin fold thickness (P < 0.001, 95% CI -0.12 to -0.04) showed significant association with the dialysis malnutrition score.

Conclusion: Mild-to-moderate malnutrition was common in both HD and PD patients. PD, sex, number of dialysis years, average hand grip strength, and average skinfold thickness were found to affect the dialysis malnutrition score.

## Introduction

Malnutrition and protein-energy wasting (PEW) are significant concerns in patients with end-stage renal disease (ESRD) undergoing hemodialysis (HD) and peritoneal dialysis (PD) [1]. Recent findings have strongly linked malnutrition and PEW with decreased quality of life and increased morbidity and mortality rates [2]. The International Society of Renal Nutrition and Metabolism (ISRNM) defines PEW as “the state of decreased body stores of protein and energy fuels, i.e., body protein and fat." PEW is a multifactorial syndrome characterized by reduced body protein stores (loss of muscle) and energy reserves (loss of fat). Clinically, PEW is strongly associated with adverse outcomes in chronic kidney disease (CKD) and dialysis populations, including increased risk of hospitalization, higher rates of infection, and elevated mortality [3].

Globally, the prevalence of PEW is increasing at an alarming rate in patients undergoing dialysis. A recent meta-analysis of 90 studies involving 16,434 dialysis patients from 34 countries estimated that the prevalence of PEW ranges from 28% to 54% [4]. In Saudi Arabia, there is limited research on the prevalence of PEW in dialysis patients. A cross-sectional study conducted in Jeddah among hemodialysis patients found that approximately 48.7% of ESRD patients were moderately malnourished and 6.3% were severely malnourished [5]. Another study, which used Patient-Generated Subjective Global Assessment (PG-SGA) in HD patients, showed that 43.7% were malnourished [6]. In another study in Saudi Arabia, 51.7% of HD patients had moderate malnutrition, and 2.6% had severe malnutrition [7]. Despite the available evidence highlighting the prevalence of malnutrition, PEW tend to go unnoticed and untreated in a range of healthcare settings. Lack of awareness, combined with insufficient knowledge and training, could pose significant challenges to effectively caring for patients.

Recent studies have indicated that various factors contribute to protein-energy wasting. Anemia, metabolic stress, increased nutrient requirements, poor appetite, decreased dietary intake, loss of nutrients during dialysis, inflammation, and other comorbidities, such as gastrointestinal disease, cardiovascular disease, diabetes, infections, and sepsis, are the principal factors that can contribute to malnutrition [1,8].

Nutritional scoring systems have become highly recommended practical tools for diagnosing malnutrition in patients undergoing dialysis [9]. Subjective Global Assessment (SGA) is an assessment tool for nutritional status that refers to an overall evaluation of a patient's history and physical examination. SGA is a valid and reliable tool that predicts morbidity and mortality associated with malnutrition [10]. The Dialysis Malnutrition Score (DMS) is a modified version of the SGA (M-SGA), with additional parameters of dialysis duration, and is reported to be superior to the conventional SGA [9]. The inclusion of dialysis duration and dialysis related parameters in the DMS enhances its sensitivity to detect malnutrition in dialysis patients, making it more specific and informative than the conventional SGA for this population. In previous Saudi Arabian studies, nutritional assessment methods varied; for example, one study used the PG-SGA to measure patients’ nutritional status, while another utilized the DMS as a screening tool. However, previous studies lacked important information such as the percentage of urea reduction ratio (URR) and Kt/V. Furthermore, these studies did not include relevant biochemical parameters or intradialytic weight gains.

Studies assessing nutritional status and analyzing sociodemographic, anthropometric, laboratory, and dietary factors and nutrient intake in HD and PD patients in Saudi Arabia are also scarce. This study aimed to assess the nutritional status of HD and PD patients and to determine the factors associated with malnutrition.

This abstract was previously presented as a conference abstract at the Nutrition 2025 Conference (American Society for Nutrition, Florida, USA). The abstract was published in *Current Developments in Nutrition* (available from: https://cdn.nutrition.org/article/S2475-2991(25)01629-4/fulltext).

## Materials and methods

Study design and data collection

This cross-sectional prospective observational study was conducted at the HD unit and PD clinic of King Fahad Armed Forces Hospital in Jeddah, Saudi Arabia. The data were collected between November 2022 and June 2023.

Subjects

The inclusion criteria were as follows: i) adults aged between 18 and 65 years; ii) undergoing HD three times per week for at least four hours per session and patients undergoing automated peritoneal dialysis (APD); iii) on dialysis for at least six months; iv) not on enteral and parenteral feeding; and v) ability to stand. The exclusion criteria were as follows: i) any physical, mental, or psychiatric disease(s); ii) amputation; iii) presence of bacterial infections or chronic viral infections such as human immunodeficiency virus (HIV) and hepatitis B or C; v) history of hospitalization, peritonitis, or central line-associated bloodstream infection in the past three months; and vi) communication disability. Based on these criteria, 398 HD and 90 PD patients were screened, and 200 HD and 45 PD patients were recruited (total = 245 eligible participants). Ethical approval was obtained from the Research Ethics Committee (REC 532) of the King Fahad Armed Forces Hospital prior to the commencement of the study. Patients who met the inclusion criteria provided written informed consent before participating, and their anonymity was maintained. The study was conducted in accordance with the principles of the Declaration of Helsinki.

Sample size calculation

The sample size was calculated using Epi Info™ software version 7.2 (Centers for Disease Control and Prevention, Atlanta, USA). The calculation was based on an estimated dialysis population of approximately 500 patients per year. An expected prevalence of 50% was used to yield the maximum sample size, with a 95% confidence level and a 5% margin of error. After applying the finite population correction, the final sample size required was 217 participants. This approach follows the standard method for estimating proportions in cross-sectional studies and is consistent with recent methodological recommendations emphasizing transparent reporting of parameters used in sample size estimation [11-13].

Although the minimum required sample size was 217 participants, all 245 eligible patients identified during the recruitment period were included to enhance the precision and representativeness of the study findings.

Anthropometric measurements

The heights of all patients were measured in centimeters while they were barefoot. Dry body weight and post-dialysis weight were measured in kilograms for the HD patients. Body mass index (BMI) was calculated, using the formula weight (kg)/height² (m) for both HD and PD patients. The patients were classified into BMI categories as follows: underweight (<18.5 kg/m^2^), normal weight (18.5-<25 kg/m^2^), overweight (25-<30 kg/m^2^), and obese (≥30 kg/m^2^). Intradialytic weight gain (kg) in the HD patients was also recorded. Triceps skinfold thickness (TSF) was measured using a Harpenden skinfold caliper on the non-fistula arm for HD patients and on the right arm for PD patients. Mid-arm circumference (MAC) was measured twice on the non-access arm for all participants using a flexible, non-stretchable measuring tape, and the average of the two readings was recorded. Mid-arm muscle circumference (MAMC) was calculated using the formula: MAMC = MAC (cm) - (0.314 × TSF(mm)). All anthropometric measurements were performed by a trained clinical dietitian. 

Hand grip strength

Hand grip strength (HGS) was measured with an electronic HGS dynamometer on the non-fistula side for HD after a dialysis session [14] using CAMRY mechanical dynamometers (Sammons Preston, Masan, Korea) with a precision of 0.5 kg. The participants were instructed to adjust the dynamometer to ensure a comfortable fit for optimal performance. Trained dietitians advised the subjects to grip the dynamometer with maximum strength and to apply as much handgrip pressure as possible. HGS was measured in the non-fistula hand after the HD session and in the right arm of PD patients. Patients were asked to stay in a seated position with their arms in adduction, their elbow flexed at a 90-degree angle, and the forearm and wrist positioned neutrally. Two trials were measured (kg) with a minimum rest period of one minute between each trial; the average of the two was included in the analysis.

Biochemical measurements

Laboratory parameters were measured monthly during pre-dialysis sessions for HD patients and during clinic visits for patients with PD. Patients were required to fast for 12 hours prior to blood collection by trained hospital staff. Laboratory parameters included serum albumin, hemoglobin, sodium, potassium, phosphate, total protein, and transferrin levels. Dialysis adequacy was assessed separately in each group, HD and PD, using Kt/V and URR. These measurements were not intended for comparison between modalities. This included Kt/v and the URR. In the calculation for KT/V, K represents the dialyzer clearance of the urea, where T is the dialysis time and V is the volume of distribution of urea, which is approximately equal to the patient’s total body water. URR was calculated using the following formula: URR calculator (pre-dialysis urea level - post-dialysis urea level) / pre-dialysis urea level x 100.

Dietary intake

Information about patients’ dietary intake was collected through structured face-to-face interviews by trained clinical dietitians using a 24-hour dietary recall on two non-consecutive days: one weekday and one weekend day. [9] This approach was chosen to better reflect habitual dietary intake and improve accuracy. Visual aids and standardized portion size charts were used to assist patients in estimating food quantities. Each interview typically lasted 30-60 minutes. A total of 237 out of 245 patients agreed to complete the two-day dietary recall; the remaining participants declined the dietary interviews but continued to participate in all other study procedures. For PD patients, dietary intake was recorded without the need to distinguish between dialysis and non-dialysis days, as PD is performed continuously on a daily basis. In contrast, for HD patients, data collection was carried out during dialysis sessions, and patients were asked to recall their food intake from the previous day. Therefore, the recorded intake for most HD patients likely reflected non-dialysis days. Dietary data were manually entered into the online nutrition analysis software “Nutritics” (version 5.09, Dublin, Ireland), which provided nutrient values including energy (kcal), protein (g), sodium (mg), potassium (mg), and phosphorus (mg). Nutrient adequacy was evaluated based on the Kidney Disease Outcomes Quality Initiative (KDOQI) dietary recommendations [15].

Dialysis malnutrition score

The DMS is a simple and dynamic tool for assessing malnutrition in ESRD patients. It is a five-point scale modified quantitative SGA, which includes the two major components of medical history and physical evaluation. The DMS was performed by trained clinical dietitians conducting interviews. Each part of the DMS was subjectively rated on a scale of 1 to 5, where 1 = normal nutrition, 2 = 4 moderate malnutrition, and 5 = severe malnutrition [9].

Demographic and medical history questionnaire

A general questionnaire was provided to the recruited patients to collect general information regarding their socioeconomic backgrounds. It included their level of education, marital status, employment status, monthly income, accommodation type, tobacco use, type of dialysis, number of dialysis years, presence of comorbid diseases, and medications taken.

Statistical analysis

Statistical analyses were performed using IBM SPSS Statistics for Windows, version 27.0 (released 2019, IBM Corp., Armonk, NY). Categorical variables are presented as frequencies and relative frequencies. Numerical data are presented as mean and standard deviation. Simple linear regression and multiple linear regression models were applied using the backward stepwise method to study factors affecting the dialysis malnutrition score. Differences were considered statistically significant at p ≤ 0.05.

## Results

Selection of eligible participants

**Figure 1:**
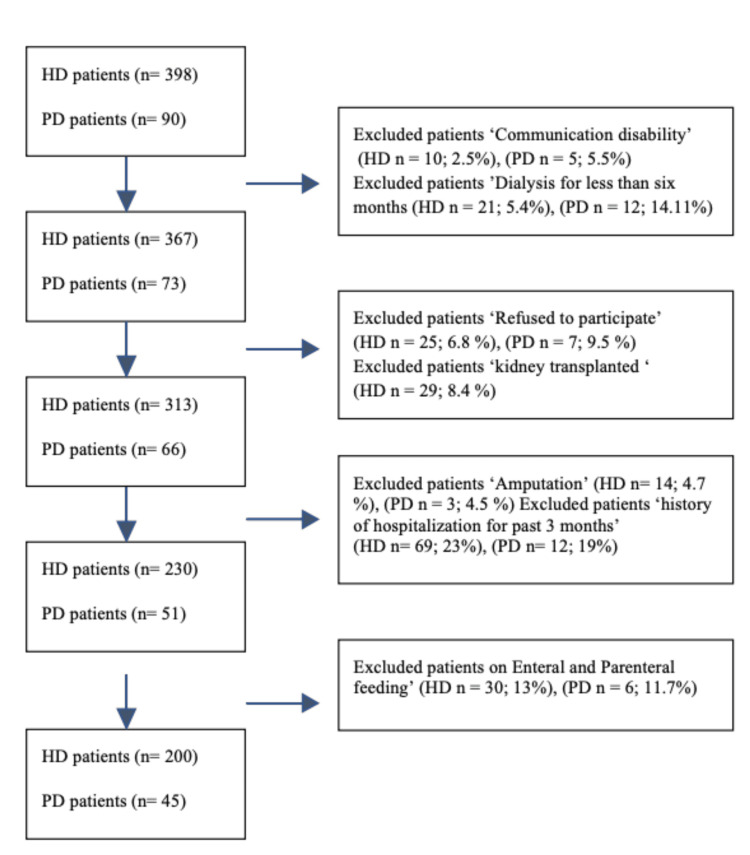
Selection of eligible participants Flow chart showing selection of eligible participants. HD: hemodialysis; PD: peritoneal dialysis

Of the patients, 10 HD patients (2.5%) and five PD patients (5.5%) were excluded due to communication disability. In addition, 21 HD patients (5.4%) and 12 PD patients (14.11%) were excluded because they had been on dialysis for less than six months. Twenty-five HD (6.8%) and seven PD patients (9.5%) refused to participate and were excluded. Moreover, 29 HD patients (8.4%) had received a transplant. Fourteen HD patients (4.7%) and three PD patients (4.5%) who had amputation were also excluded. Patients who had been hospitalized during the past three months were excluded as well: 69 HD patients (23%) and 12 PD patients (19%). In addition, 30 HD patients (13%) and six PD patients (11.7%) receiving enteral or parenteral feeding were excluded. Figure 1 shows the selection of eligible participants. Based on the study’s inclusion criteria, 200 HD and 45 PD patients were recruited, with participation rates of 50% for both groups.

Sociodemographic characteristics and medical history for dialysis patients

A total of 245 patients were included in the analysis of sociodemographic characteristics and comorbidity (Table 1). Among them, 200 patients (81.6%) were on HD, and 45 patients (18.4%) were on PD. The mean age of the patients was 56.78 ± 16.12 years, with 53.9% aged over 55 years. Gender distribution was almost equal, with 121 males (49.4%) and 124 females (50.6%). Regarding educational level, 158 patients (64.5%) had an education level below high school, whereas 58 (23.7%) had a diploma or completed high school. The majority of patients, 160 (65.3%), were married, and 214 (87.3%) were unemployed. In terms of monthly income, 97 patients (39.6%) reported earning less than 4,000 SR, while 26.1% reported an income between 4,000 and 6,000 SR. In addition, 14.3% of patients were smokers. The mean duration of dialysis treatment per year was 4.87 ± 4.81 years, while the mean weekly dialysis was 3.81 per week (SD = 1.87). As for comorbidities, 152 patients (62.0%) had diabetes mellitus, 225 (91.8%) had hypertension, 66 patients (26.9%) had cardiac diseases, and 14 patients (5.7%) had a history of cerebral vascular accident.

**Table 1:** Sociodemographic and clinical characteristics for dialysis patients (N = 245) N: number of patients; %: percentage; HD: hemodialysis; PD: peritoneal dialysis; SD: standard deviation; DM: diabetes mellitus; HTN: hypertension; CVA: cerebral vascular accident

Sociodemographic characteristics	
Gender	N (%)
Male	121 (49.4%)
Female	124 (50.6%)
Age (years), Mean ± SD	56.78 ± 16.12
<= 55 years	113 (46.1%)
> 55 years	132 (53.9%)
Education level	N (%)
< High school	158 (64.5%)
Diploma or high school	58 (23.7%)
Bachelor	28 (11.4%)
Postgraduate	1 (0.4%)
Marital status	N (%)
Single	30 (12.2%)
Married	160 (65.3%)
Divorced	13 (5.3%)
Widow	42 (17.1%)
Employment status	N (%)
Unemployed	214 (87.3%)
Employed	31 (12.7%)
Income	N (%)
< SR 4000	97 (39.6%)
SR 4000-6000	64 (26.1%)
SR 6001-10000	53 (21.6%)
SR 10001-15000	9 (3.7%)
> SR 15000	22 (9%)
Accommodation type	N (%)
Rent	99 (40.4%)
Owned	146 (59.6%)
Tobacco use	N (%)
Non-smoker	210 (85.7%)
Smoker	35 (14.3%)
Clinical characteristics	
Type of dialysis	N (%)
HD	200 (81.6%)
PD	45 (18.4%)
Comorbidities	N (%)
DM	152 (62%)
HTN	225 (91.8%)
Cardiac diseases	66 (26.9%)
CVA	14 (5.7%)
Hyperparathyroidism	14 (5.7%)
Hypothyroidism	18 (7.3%)
Others	110 (44.9%)
Parameter	Mean ± SD
Dialysis per week	3.81 ± 1.87
Number of dialysis years	4.87 ± 4.81
Number of medications used	10.1 ± 3.02

Anthropometric measurements

The mean dry weight of the patients was 67.85 ± 18.73 kg, while the mean intradialytic weight gain (IDWG) was 1.61 ± 1.26 kg. The mean height of the patients was 160.35 ± 9.85 cm, and the mean BMI was 26.17 ± 6.18 kg/m^2^. Based on the BMI classification, 17 patients (7.0%) were underweight, 72 patients (29.5%) were overweight, and 56 patients (23.0%) were obese. Anthropometric measurements are summarized in Table 2.

**Table 2:** Anthropometric measurements and weight status for dialysis patients IDWG: intradialytic weight gain; BMI: body mass index; SD: standard deviation; N: number of patients, %: percentage

Mean ± SD	Anthropometric measurements
67.85 ± 18.73	Dry weight (kg)
1.61 ± 1.26	IDWG (kg)
160.35 ± 9.85	Height (cm)
26.17 ± 6.18	BMI (kg/m2)
N (%)	BMI category
17 (7.0%)	Underweight
99 (40.6%)	Healthy weight
72 (29.5%)	Overweight
56 (23.0%)	Obese

Laboratory measurements

The mean albumin of the dialysis patients was 37.17 ± 4.57 g/L; the mean sodium was 134.22 ± 3.93 mmol/L; the mean potassium was 5.18 ± 3.87 mmol/L; the mean phosphorus was 1.66 ± 0.64 mmol/L; and the mean total protein was 67.55±7.1 g/L, as shown in Table 3

**Table 3:** Laboratory measurements for dialysis patients SD: standard deviation; URR: urea reduction ratio; Kt/v: k = clearance the amount of the urea dialyzer can remove, T = time, V = volume

Mean ± SD	Laboratory measurements
37.17 ± 4.57	Albumin (g/L)
109.89 ± 19.22	Hemoglobin (g/L)
134.22 ± 3.93	Sodium (mmol/L)
5.18 ± 3.87	Potassium (mmol/L)
1.66 ± 0.64	Phosphorus (mmol/L)
67.55 ± 7.1	Total protein (g/L)
1.63 ± 0.33	Transferrin (g/L)
74.27 ± 8.42	URR
1.52 ± 0.65	Kt/v (ml/min)

Dietary intake

Regarding dietary and nutritional components, the mean energy intake was 1064 ± 741.7 kcal/day, and protein was 49.6 ± 24.3 g/day. Sodium intake showed considerable variability, with a mean of 1658.8 ± 1294 mg/d; potassium mean intake was 1212.3 ± 618.7 mg/day; and mean phosphate intake was 590.8 ± 286.2 mg/d. Regarding nutrient needs in dialyzed patients, only six out of 129 patients (5%) under the age of 60 met the recommended daily energy intake (≥35 kcal/kg/day), and only four out of 108 patients (4%) aged ≥60 met the recommended intake (30-35 kcal/kg/day). For protein intake, 31 out of 237 patients (13%) achieved the recommended daily protein intake for dialysis patients (≥1.2 g/kg/day), as shown in Table 4.

**Table 4:** Dietary intake for dialysis patients SD: Standard deviation, *KDOQI (kidney disease outcomes quality initiative) guidelines (2000)

Mean ± SD	Dietary intake (N = 237)
1064 ± 741.7	Energy (Kcal/d)
130.6 ±57.6	Carbohydrate (g/d)
49.6 ± 24.3	Protein (g/d)
31.4 ±17.8	Fat (g/d)
1212.3 ± 618.7	Potassium (mg/d)
1658.8 ± 1294	Sodium (mg/d)
590.8 ± 286.2	Phosphorus (mg/d)
N (%)	*Energy intake <60 years (n=129)
6(5%)	35 kcal/kg/d
123(95%)	<35 kcal/kg/d
N (%)	≥ 60 years (n=108)
4 (4%)	30-35 kcal/kg/d
104 (96%)	<30-35 kcal/kg/d
N (%)	*Protein intake (n=237)
31 (13%)	≥1.2 gm/kg/d
206 (87%)	<1.2 gm/kg/d

Dialysis Malnutrition Scores

The nutritional status of the patients is shown in Table 5. Based on the DMS, 95.10% of the patients had mild malnutrition, 2.04% had moderate malnutrition, and 2.86% were well-nourished.

**Table 5:** Dialysis Malnutrition Scores N: number of patients, %: percentage

Malnutrition group	N %
Well-nourished (score of 0 to 7)	7 (2.86%)
Mild malnutrition (score of > 7 to < 21)	233 (95.10%)
Moderate malnutrition (score of ≥ 21 to < 35)	5 (2.04%)
Severe malnutrition (score of 35)	0 (0%)

Simple and multiple linear regression for factors affecting the DMS 

A multiple linear regression model was developed using the backward stepwise method to study the factors affecting DMS. PD patients had a higher DMS than those undergoing HD by an average of 1.65 (P = 0.023, 95% CI = 0.23-3.07). Regarding gender, as compared to male patients, female patients had a lower DMS by an average of -1.94 (P < 0.001, 95% CI = -2.95 to -0.93). Regarding income, as compared to patients who had less than 4000 SR monthly income, those who had between 4000 and 6000 SR monthly income had lower DMS by an average of -1.27 (P = 0.021, 95% CI = -2.35 to -0.20). There was a statistically significant association between the number of dialysis years and DMS; for every unit increase in the number of dialysis years, there was an average of 0.15-unit increase in the DMS (P = 0.001, 95% CI = 0.07-0.24). The average hand grip strength was significantly associated with DMS; for every unit increase in the average hand grip, there was a -0.07 unit decrease in the DMS (P < 0.001, 95% CI = -0.10 to -0.03). The mean TSF was statistically associated with DMS; for every unit increase in the mean TSF, there was a -0.08 unit decrease in the DMS (P < 0.001, 95% CI = -0.12 to -0.04). Table 6 illustrates the simple and multiple linear regression models used to identify the factors affecting DMS.

**Table 6:** Simple and multiple linear regression for factors affecting the Dialysis Malnutrition Score Ref: reference category; * significant as a p-value ≤ 0.05; CI: confidence interval; DM: diabetes mellitus; CVA: cerebrovascular accident; Kt/v: k = clearance the amount of urea dialyzer can remove, t = time, v = volume; IDWG: intradialytic weight gain; BMI: body mass index; TSF: triceps skinfold thickness

	Univariate analysis			Multivariable analysis		
	Coefficient	P-value	95% C.I.	Coefficient	P-value	95% C.I.
Type of dialysis						
Hemodialysis	Ref.					
Peritoneal dialysis	0.99	0.095	-0.17 to 2.15	1.65	0.023*	0.23 to 3.07
Gender						
Male	Ref.					
Female	-0.27	0.551	-1.18 to 0.63	-1.94	<0.001*	-2.95 to -0.93
Age	0.04	0.007*	0.01 to 0.07			
Marital status						
Single	Ref.					
Married	0.49	0.494	-0.92 to 1.89	-	-	-
Divorced	-1.35	0.258	-3.69 to 0.99	-	-	-
Widow	0.67	0.437	-1.02 to 2.35	-	-	-
Educational level						
Less than high school	Ref.					
Diploma or high school	-0.85	0.114	-1.9 to 0.21	-0.68	0.199	-1.73 to 0.36
Bachelor or postgraduate	-2.85	<0.001*	-4.23 to -1.46	-1.49	0.074	-3.13 to 0.15
Employment status						
Unemployed	Ref.					
Employed	-1.61	0.019*	-2.96 to -0.27	-	-	-
Income						
Ref.					
SR 4000-6000	-1.03	0.075	-2.16 to 0.1	-1.27	0.021*	-2.35 to -0.20
SR 6001-10000	-0.25	0.68	-1.45 to 0.95	-0.27	0.626	-1.36 to 0.82
SR 10001-15000	-1.85	0.138	-4.29 to 0.6	-1.36	0.268	-3.78 to 1.05
>SR 15000	-1.67	0.048*	-3.33 to -0.02	-1.17	0.177	-2.87 to 0.53
Comorbidities (DM)						
No	Ref.					
Yes	0.02	0.968	-0.91 to 0.95	-	-	-
CVA						
No	Ref.					
Yes	0	0.997	-1.95 to 1.94	-	-	-
Number of dialysis years	0.2	<0.001*	0.1 to 0.29	0.15	0.001*	0.07 to 0.24
Kt/v, (mL/min)	0.33	0.373	-0.4 to 1.07	-	-	-
IDWG (kg)	-0.41	0.025*	-0.76 to -0.05	-	-	-
BMI (kg/m^2 ^)	-0.17	<0.001*	-0.24 to -0.1	-	-	-
Albumin (g/l)	-0.04	0.396	-0.14 to 0.06	-	-	-
Energy (kcal/d)	0	0.512	0 to 0	-	-	-
Protein (g/d)	-0.01	0.151	-0.03 to 0	-	-	-
Average hand grip (kg)	-0.06	<0.001*	-0.09 to -0.03	-0.07	<0.001*	-0.10 to -0.03
Average TSF (mm)	-0.12	<0.001*	-0.15 to -0.08	-0.08	<0.001*	-0.12 to -0.04

## Discussion

The management of patients with ESRD is challenging. Despite advances in renal replacement therapies, such as HD and PD, and a more viable option for kidney transplantation, patients still develop complications, including malnutrition. Dialysis is a catabolic procedure that results in the loss of amino acids and vitamins and disturbs glucose metabolism, depending on the dialysates used [16]. Patients undergoing thrice-weekly dialysis sessions can experience an annual loss of 2 kg in their lean body mass [5,17]. Hence, the number of PEW cases is increasing among dialysis patients. Various factors, such as increased nutrient requirements, anorexia, altered taste sensation, emotional distress, gastrointestinal symptoms, and catabolic metabolism, contribute to PEW [18]. Evaluating nutritional status is crucial for improving the quality of life and preventing associated complications in patients undergoing dialysis treatment. In this study, DMS and HGS, along with dietary intake and anthropometric and biochemical data, were used as screening tools for malnutrition. The DMS was enhanced by incorporating factors such as functional capacity, comorbidities, decreased fat stores, and signs of muscle wasting, making it an effective tool for the comprehensive evaluation of nutritional status [19].

Malnutrition is very common, but predominantly mild malnutrition among our patients. This predominance of mild malnutrition may be explained by reduced appetite on dialysis days, low-grade chronic inflammation, and variable adherence to dietary recommendations. PD, long-vintage renal replacement therapy, HGS and TSF, and non-adherence to nutritional requirements significantly affected DMS. The study's findings are significant, with the majority of participants showing mild malnutrition (95%) and a minority showing moderate malnutrition (2.04%). These results are consistent with those of previous studies conducted in Jeddah. In 2009, Alharbi et al. found that 54.3% of hemodialysis patients were moderately malnourished, which is the first study to identify malnutrition in Saudi Arabia. Kutbi et al. discovered that approximately 43.7% of patients were either mildly or moderately malnourished [6], and Azzeh et al. found that 51.7% had mild or moderate malnutrition [7]. These studies shared common factors that might correlate with malnutrition, including an increasingly aging population, low education level, and unemployment. Alharbi et al. and Azzeh et al. found that increased dialysis time leads to a greater risk of malnutrition [5,7]. Similar findings were observed in the present study. The findings also suggest that males are at a higher risk of malnutrition than females, but this does not correlate with Alharbi et al.'s findings [5]. Male gender remained significantly associated with higher malnutrition scores even after adjusting for age, comorbidities, and dialysis modality. This could be due to the difference in the number of males in their study. Hand grip strength measurement is an inexpensive, simple, fast, and reliable method for evaluating muscle strength and malnutrition [19]. Similar to the findings of Azzeh et al., we found a significant inverse association between hand grip strength and malnutrition [7]. Patients with ESRD who undergo dialysis often struggle to adhere to the KDOQI dietary recommendations, which results in their inability to meet energy and macronutrient needs [15]. This is observed in our findings, where only 10 out of 237 patients met the daily energy requirement, and 31 of them met the protein needs.

Most studies conducted in Saudi Arabia focused on HD patients. This study is the first to screen for malnutrition among people undergoing peritoneal dialysis in the Kingdom of Saudi Arabia. Patients with PD had higher DMS scores than those with HD. This may be related to continuous protein loss through PD effluent and the metabolic stress associated with peritoneal dialysis. Clinically, these findings highlight the importance of enhanced nutritional surveillance and more aggressive dietary interventions for PD patients. Our sample population was representative, based on sample power calculations. A trained clinical dietitian conducted anthropometrics and comprehensive nutritional assessments and collected other data, such as sociodemographic and dietary intake.

Limitations of the study

This study has several limitations that should be considered when interpreting the findings. First, dietary assessment was based on two non-consecutive 24-hour recalls (one on a dialysis day and one on a non-dialysis day), which may not fully capture long-term dietary habitual intake. Additional tools, such as multiple recalls or food frequency questionnaires (FFQs), could provide complementary information and enhance the assessment of usual dietary patterns. Second, the PD group had a relatively small sample size, which may limit the statistical representativeness of the findings. Third, potential confounding factors, such as dietary adherence, physical activity, and socioeconomic status, were not fully controlled in the analysis. Finally, the single-center design may reduce the generalizability of the results to the broader dialysis populations

## Conclusions

People with ESRD who are placed on HD/PD commonly experience malnutrition. This might be related to the factors that affect dialysis malnutrition disease score: age, PD, income, long vintage of renal replacement therapy, hand grip strength, and skinfold thickness. Hence, taking into consideration these factors as part of the nutritional assessment might be one of the keys to limiting the occurrence of malnutrition. Nevertheless, understanding these factors might lead to better strategies to increase dietary recommendation adherence. However, more investigation needs to be conducted with a larger sample size. 
